# A Drought Resistance-Promoting Microbiome Is Selected by Root System under Desert Farming

**DOI:** 10.1371/journal.pone.0048479

**Published:** 2012-10-31

**Authors:** Ramona Marasco, Eleonora Rolli, Besma Ettoumi, Gianpiero Vigani, Francesca Mapelli, Sara Borin, Ayman F. Abou-Hadid, Usama A. El-Behairy, Claudia Sorlini, Ameur Cherif, Graziano Zocchi, Daniele Daffonchio

**Affiliations:** 1 Dipartimento di Scienze per gli Alimenti, la Nutrizione e l’Ambiente, Università degli Studi di Milano, Milan, Italy; 2 Laboratoire Microorganismes et Biomolécules Actives, Université Tunis El Manar, Tunis, Tunisia and Laboratoire Biotechnologie et Valorisation des Bio-Géo Ressources, Institut Supérieur de Biotechnologie, Université de La Manouba, Sidi Thabet, Ariana, Tunisia; 3 Dipartimento di Scienze Agrarie e Alimentari- Produzione, Territorio, Agroenergia; Università degli Studi di Milano, Milan, Italy; 4 Department of Horticulture, Ain Shams University, Cairo, Egypt; Argonne National Laboratory, United States of America

## Abstract

**Background:**

Traditional agro-systems in arid areas are a bulwark for preserving soil stability and fertility, in the sight of “reverse desertification”. Nevertheless, the impact of desert farming practices on the diversity and abundance of the plant associated microbiome is poorly characterized, including its functional role in supporting plant development under drought stress.

**Methodology/Principal Findings:**

We assessed the structure of the microbiome associated to the drought-sensitive pepper plant (*Capsicum annuum* L.) cultivated in a traditional Egyptian farm, focusing on microbe contribution to a crucial ecosystem service, i.e. plant growth under water deficit. The root system was dissected by sampling root/soil with a different degree of association to the plant: the endosphere, the rhizosphere and the root surrounding soil that were compared to the uncultivated soil. Bacterial community structure and diversity, determined by using Denaturing Gradient Gel Electrophoresis, differed according to the microhabitat, indicating a selective pressure determined by the plant activity. Similarly, culturable bacteria genera showed different distribution in the three root system fractions. *Bacillus* spp. (68% of the isolates) were mainly recovered from the endosphere, while rhizosphere and the root surrounding soil fractions were dominated by *Klebsiella* spp. (61% and 44% respectively). Most of the isolates (95%) presented *in vitro* multiple plant growth promoting (PGP) activities and stress resistance capabilities, but their distribution was different among the root system fractions analyzed, with enhanced abilities for *Bacillus* and the rhizobacteria strains. We show that the *C. annuum* rhizosphere under desert farming enriched populations of PGP bacteria capable of enhancing plant photosynthetic activity and biomass synthesis (up to 40%) under drought stress.

**Conclusions/Significance:**

Crop cultivation provides critical ecosystem services in arid lands with the plant root system acting as a “resource island” able to attract and select microbial communities endowed with multiple PGP traits that sustain plant development under water limiting conditions.

## Introduction

The “reverse desertification” includes a series of interventions aimed to sustain soil stability and productivity in arid lands, providing tools and strategies to support crop production for human feeding while preserving biodiversity and counteracting climate changes. Desert farming represents a strategy to protect soil fertility and aims at gaining arable land at expenses of desert soil, subjected to low resources landscape [1]. Traditional and more technologically efficient desert farming systems are well established in North Africa and their spread represents an impellent necessity to provide food for the increasing world population that will rapidly reach 9 billion people in few decades [2]. Desert farming primarily relies on irrigation in an ecosystem where water is a limiting and often polluted resource. Water stress is a primary cause of crop losses, reducing average yields by more than 50% [3]. Such a decrease in productivity is attributable to a direct negative effect of water scarcity on plant physiology. Despite the recognized importance of root associated microorganisms for plant growth and health, few studies are available on how desert farming affects the diversity of the crop associated-microbiome and whether the selected microorganisms still retain plant growth abilities to sustain plant development under water limiting conditions [4]. In particular, it is poorly explored whether desert farming may promote the selection of microbes capable of enhancing a key primary ecosystem service like plant tolerance to drought.

In the family *Solanaceae*, *Capsicum annum* L. is one of the horticulture plants most sensitive to water stress [5], [6]. Pepper has great economic, agricultural and food relevance, and despite it is largely cultivated where climatic conditions are generally characterized by high temperatures and scarce water availability [7], it requires a relatively high water supply during the whole crop life cycle to obtain high yield productivity [5], [8], [9], [10], [11]. Pepper has gained the role of a model plant in physiology studies, like those conducted on the effects that plant growth promoting (PGP) bacteria have in increasing the plant resistance to stress conditions such as salinity [12], [13], [14], [15], [16]. Nevertheless, little information is available either about the distribution and diversity of the autochthonous PGP microbiome of pepper cultivated in arid lands, or the potential of the associated PGP bacteria in directly promoting plant development through a stimulation of plant drought tolerance.

Therefore, this study is aimed to assess the impact of desert farming on plant-microbe association in pepper cultivated in arid conditions. We aimed to assess the diversity and topological repartition of bacteria in the pepper root system grown under desert farming and investigate whether under such a crop management practice the root system enriches bacteria capable of supporting the plant resistance to drought and water stress.

With this aim we adopted both culture-independent and -dependent approaches. Cluster analysis was applied to DGGE (Denaturing Gradient Gel Electrophoresis) to dissect the structure and the composition of the microbiome associated to pepper endosphere, rhizosphere and root surrounding soil in comparison to unvegetated soil (bulk). A large collection of isolates from different fractions of the plant root system was established and screened *in vitro* for PGP activities. The rhizo-competence of the bacterial strains was evaluated through an adhesion assay on both *Arabidopsis thaliana* and pepper rhizoplane. Finally we assessed the capacity of selected strains to support plant growth under water deficiency.

We demonstrated that the application of desert greening techniques in arid lands generate hotspots of microbial diversity in the rhizosphere of plants. These techniques include a virtuous use of water for irrigation, field fertilization with organic fertilizers originating from residues of crops and animal manure and other similar traditional agricultural management practises. Furthermore we documented that plant rhizosphere and endosphere are repository for selected and specialized microbial populations, able to promote plant growth under drought. Thus, desert farming hampers desertification by establishing fertility islands and allows to achieve crop yields despite the adverse environmental conditions.

## Results

### Variability of the Bacterial Community Structure as Revealed by Community Fingerprinting

**Figure 1:**
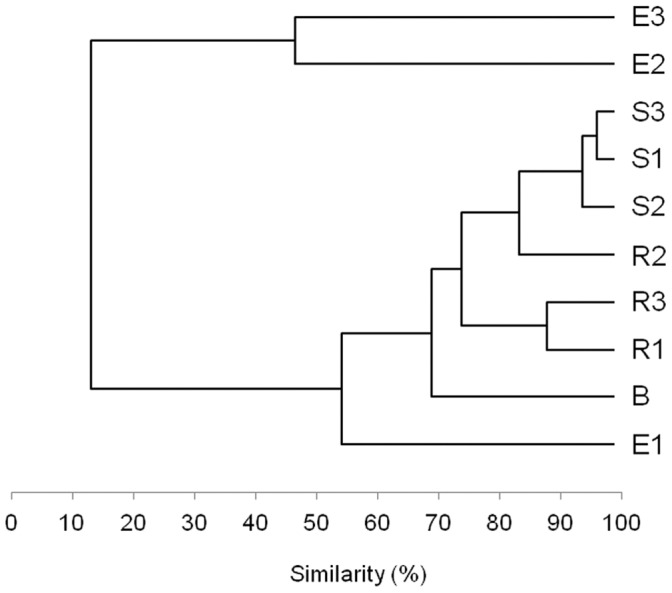
Cluster analysis of total microbial communities according to 16S rRNA DGGE profiles. The cluster analysis of the plot line was obtained from 16S rRNA PCR-DGGE bacterial community profiles, according to Pearson correlation. The analyzed fractions were root tissues (E), rhizosphere (R), root-surrounding soil (S) and bulk soil (B) of three replicate plants of pepper.

A 16S rRNA gene PCR-DGGE analysis was performed to explore the structure of the microbial communities associated to the pepper root system. The rhizosphere (R), composed of the soil particles tightly adhering to the rhizoplane, the root surrounding soil (S), composed of the soil particles not attached to the root system, and surface sterilized root tissues (E, endosphere) were compared to the non cultivated soil (B, bulk soil) (Fig. S1 and 1). While all soil fractions resulted inhabited by a complex microbiome, represented by a multiple band pattern, the pepper endosphere was represented by a restricted community (Fig. S1). Cluster analysis of the DGGE band profiles revealed a sharp difference in the microbial community structure associated to the different fractions (Fig. 1). The composition of the microbiome associated to the soil fractions R and S hosting the plant clearly differed from the arid root-free soil, indicating that farming practices profoundly affect soil microbiome structure (Fig. 1). A rhizosphere effect could be also observed since the closeness of the root tissues determined a change of bacterial community structure in the R samples respect to the S samples. The pepper endosphere resulted rather different from the soil-borne fractions by approximately 50% of the detected bands, indicating a strong selection pressure determined by the plant tissues (Fig. 1).

The dominant taxa associated with the PCR-DGGE profiles were identified by partial 16S rRNA band sequencing and their prevalence in the pepper root system and the non-cultivated arid soil was determined (Table 1). The major taxa associated to the pepper root system were affiliated to *Actinobacteria*, *Bacilli*, A*lpha*, *Beta* and *Gammaproteobacteria*. A certain taxa specificity was associated to the different fractions of the root system (Table 1). R and S fractions were dominated by *Proteobacteria* and spore forming bacteria of the genus *Bacillus* and related genera. *Actinobacteria* were retrieved only associated to plant root tissues and uncultivated root-free arid soil (Table 1). A differential repartition between the fractions was also observed for some *Proteobacteria*: *Thiobacillus* sp. was found only in the bulk soil, while some *Pseudoxanthomonas* sp. were typical of the endosphere fraction (Table 1).

**Table 1:** Phylogenetic identification and distribution of bacteria excised and sequenced from DGGE bands.

Band	Class (RDP)	Closest Relative (accession number)	%	Closest type strain or described cultivable strain (accession number)	%	Fraction of the pepper root system									
						E1	E2	E3	R1	R2	R3	S1	S2	S3	B
4	*Actinobacteria*	*Microbacterium phyllosphaerae* (HM355641)	100	*Microbacterium phyllosphaerae* (HM355641)	100	**X**									
22	*Actinobacteria*	Uncultured Bacterium (DQ129271)	97	*Nocardioides mesophilus* (EF466117)	97										**X**
1	*Bacilli*	Uncultured Bacterium (FN563205)	98	*Paenibacillus taichungensis* (FJ944633)	95	**X**	**X**		X			X	X	X	
8				*Paenibacillus gansuensis* (NR_043219)	95										
3		Uncultured Bacterium (FN563205)	97	*Paenibacillus motobuensis* (EU982886)	94			X	X	X	**X**	X	X	X	X
16	*Bacilli*	Uncultured *Paenibacillus* sp. (JX501979)	99	*Paenibacillus chitinolyticus* (AB680938)	95					**X**	X	X	X	**X**	X
20			98	*Paenibacillus chitinolyticus* (FJ944683)	96										
21	*Bacilli*	*Bacillus megaterium* (JX312633)	98	*Bacillus megaterium* (JX312633)	98	X			X	X	X	X	X	X	**X**
2	*Alphaproteobacteria*	Unclassified *Alphaproteobacterium* (AY162055)	98	*Sphingobium ummariense* (NR_044171)	98	**X**			X	X	X	X	X	X	
11	*Betaproteobacteria*	Uncultured Bacterium (HQ272664)	98	*Thiobacillus thioparus* (HM173633)	97	X	X		**X**	X	X	X	X	X	X
19	*Betaproteobacteria*	*Thiobacillus denitrificano* (EU546130)	98	*Thiobacillus denitrificano* (EU546130)	98				X	X	X	X	X	**X**	X
5	*Gammaproteobacteria*	*Rhodanobacter lindaniclasticus* (AB245366)	100	*Rhodanobacter lindaniclasticus* (AB245366)	100	**X**									
13	*Gammaproteobacteria*	*Rhodanobacter lindaniclasticus* (AB245366)	97	*Rhodanobacter lindaniclasticus* (AB245366)	97				**X**	X	X	**X**	X		
17	*Gammaproteobacteria*		99		99										
6	*Gammaproteobacteria*	*Pseudoxanthomonas ginseng isoli* (JF778717)	99	*Pseudoxanthomonas ginseng isoli* (JF778717)	99	**X**	X								
10		*Pseudoxanthomonas ginseng isoli* (JF778717)	99	*Pseudoxanthomonas ginseng isoli* (JF778717)	99				**X**	**X**	X		X	X	
15			99		99										
7	*Gammaproteobacteria*	*Pseudoxanthomonas ginseng isoli* (JF778717)	99	*Pseudoxanthomonas ginseng isoli* (JF778717)	99	**X**	X								
9	*Gammaproteobacteria*	*Dyella yeojuensis* (FN796854)	99	*Dyella yeojuensis* (FN796854)	99			**X**							
12	*Gammaproteobacteria*	Uncultured *Thermomonas* sp. (EF072902)	99	*Lysobacter pocheonensis* (EU273938)	98				**X**	X	X	X	X		
14	*Gammaproteobacteria*	Uncultured *Thermomonas* sp. (EF072902)	99	*Lysobacter pocheonensis* (EU273938)	98				**X**	X	X	X	**X**	X	
18			98		97										

Identification of the dominant bands in the PCR-DGGE fingerprinting profiles (marked in Fig. S1) and their distribution in the different fractions of the pepper root system. The codes of the different fractions of the pepper root systems are as follow: E, Endosphere; R, rhizosphere; S, root-surrounding soil; B, non-cultivated root-free arid soil. The numbers following the codes indicate the different replicates.

X: presence of the band in the DGGE profile of the indicated fraction; in bold are indicated the bands that were actually sequenced.

Sequences of bands with the same mobility in the DGGE gel are reported in the same white/grey boxes. In some cases the different bands showed slightly different sequences whit few nucleotide variations. When the variation resulted within the 3% divergence on the 16S rRNA sequence, the bands where assumed to belong to the same OTU at the 97% identity threshold, as evaluated using DOTUR [68].

### Quantitative Analysis of Bacterial Abundance

Statistically higher microbial counts were recorded for the culturable bacteria associated to R fraction in both R2A and KB media [(5.13±3.44)×10^9^ and (1.28)×10^8^ CFU g^−1^ fresh weight, respectively] in comparison to the non-cultivated arid soil [(1.28±0.72)×10^8^ and (3.74±2.64)×10^7^ CFU g^−1^ fresh weight, respectively] as shown in Table 2. While the culturable microbiome associated to S fraction showed viable counts with intermediate values between R and B fractions, significantly lower CFU [(9.62±4.53)×10^6^ and (1.92±1.07)×10^5^ CFU g^−1^ fresh weight, respectively] were observed in the pepper endosphere (Table 2). In contrast, the abundance of culturable ACC-deaminase (ACCd) bacteria showed a dramatic reduction in the non cultivated soil [(9.81±2.64)×10^4^ CFU g^−1^ fresh weight] in comparison to plant associated fractions, where bacterial counts were detected at least four order of magnitude higher (Table 2).

**Table 2:** Abundance of culturable bacteria associated to the different fractions of the pepper root system.

Fraction	Bacterial Count (CFU g^−1^ fresh weight)			N° isolates			ACCd haplotypes
	R2A	KB	ACC	R2A	KB	ACC	
E	(9.62±4.53) 10^6^	(1.92±1.07) 10^5^	(1.60±4.53) 10^8^	12	12	53	5
R	(5.13±3.44) 10^9^	(1.28±0.00) 10^8^	(2.24±4.53) 10^9^	12	12	50	8
S	(5.83±4.06) 10^8^	(2.47±1.81) 10^7^	(2.48±2.28) 10^9^	12	12	49	6
B	(1.28±0.72) 10^8^	(3.74±2.64) 10^7^	(9.81±2.64) 10^4^	12	12	51	5

The isolation was performed on different cultivation media. In the table it is reported the amount of bacterial isolates composing the strain collection associated to pepper endosphere and root-associated soil fractions. E, Endosphere; R, rhizosphere; S, root-surrounding soil; B, non-cultivated arid soil.

### Phylogenetic Analysis of Cultivable Bacteria Associated to Pepper Root System

The generated microbial collection from the root system of pepper included a total of 299 bacterial strains (Table 3). Phylogenetic affiliation was performed by 16S rRNA partial sequencing; prior to this procedure, ACCd bacteria were de-replicated by strain typing through ribosomal spacers fingerprinting in order to define the different haplotypes. (Table 2).

**Table 3:** Distribution of microbial taxa in the collection of culturable bacterial isolates associated to pepper plants.

Phylogenetic group	Genus	E (77)	R (74)	S (73)	B (75)	Species	E (77)	R (74)	S (73)	B (75)
*Firmicutes*	*Bacillus*	52	21	20	25	*B. acidiceler*			1	
						*B. altitudinis*				5
						*B. amyloliquefaciens*	1		3	2
						*B. antraci*	1			
						*B. aquimaris*				1
						*B. cereus*	3	6	2	3
						*B. endophyticus*	3	1		1
						*B. firmus*	1			
						*B. funiculus*			1	
						*B. massiliensis*			1	
						*B. megaterium*	13	7	7	13
						*B. pumilus*			1	2
						*B. subtilis*	30	5	4	1
						*B. thuringiensis*		2	1	1
	*Lysinibacillus*	2		1		*L. fusiformis*	2			
	*Paenibacillus*	23				*P. illinoisensis*	23			
*Betaproteobacteria*	*Achromobacter*		1			*A. xylosoxidans*		1		
*Gammaproteobacteria*	*Acinetobacter*		1			*A. calcoaceticus*		1		
	*Citrobacter*		3	12		*C. freundii*		3	12	
	*Klebsiella*		45	32	1	*K. oxytoca*		12		
						*K. pneumoniae*		33	32	1
	*Pseudomonas*		3	3		*P. fluorescens*		1		
						*P. koreensis*		1		
						*P. mediterranea*			2	
						*P. plecoglossicida*			1	
						*P. putida*		1		
	*Raoultella*			5		*R. planticola*			5	
*Actinobacteria*	*Cellulosimicrobium*				43	*C. cellulans*				43
	*Rhodococcus*				2	*R. fascians*				2

Numbers indicate the number of strains assigned to each genus or species, respectively.

The numbers in parentheses are the total number of isolates for each fraction.

**Figure 2:**
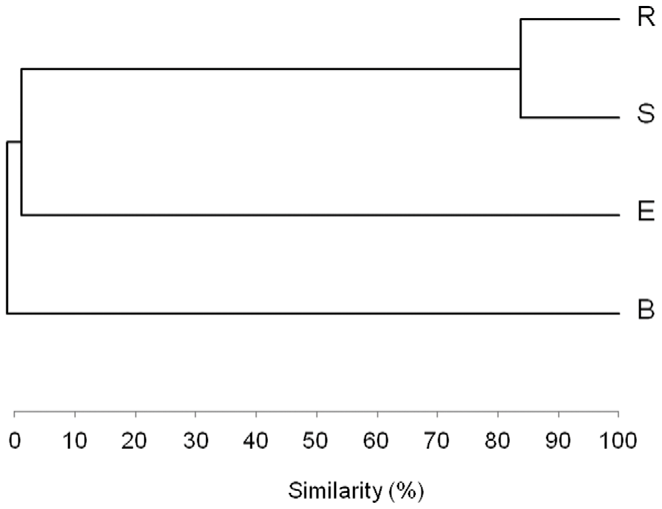
Cluster analysis of the cultivable bacteria associated to pepper fractions. The cultivable fraction of pepper-associated bacteria was compared to uncultivated soil, by performing a cluster analysis according to Pearson correlation.

Isolates were assigned to four phyla, namely *Firmicutes, Beta* and *Gamma*-subgroups of *Proteobacteria* and *Actinobacteria*, similarly to what observed by the cultivation-independent approach (Table 3). A differential distribution pattern of the major bacterial taxa among the different fractions of the pepper root system was observed (Table 3). According to cluster analysis, the composition of the cultivable community associated to R and S fractions shared a high similarity (83%), whereas that associated to the non-cultivated arid bulk soil differed significantly (Fig. 2). Despite being rather different under DGGE analysis (Fig. S2), the pepper root endosphere and the non-cultivated arid root-free soil resulted less distant according to cluster analysis (Fig. 2), presumably because of the abundance of the *Bacillus* isolates in both fractions (68% and 39%, respectively). *Bacillus, Klebsiella* and *Cellulosimicrobium* represented the most abundant genera in the bacterial collection (41%, 26% and 14%, respectively). In more detail, the pepper endosphere was dominated by the *Firmicutes* phylum and the strains were assigned to 3 genera: *Bacillus, Paenibacillus* and *Lysinibacillus,* which accounted for 68%, 30% and 3% of the isolates, respectively (Table 3 and Fig. S2). Thus E fraction was colonized by a restricted and peculiar community, as reflected by Shannon and Evenness indices (Table 4). In contrast, the R fraction showed the greatest biodiversity in terms of community structure (Table 3–4 and Fig. S2). The strains isolated from R were grouped within the *Proteobacteria* phylum (71%), comprising mainly *Gammaproteobacteria* (70%) and *Betaproteobacteria* (1%). Members of the *Gammaproteobacteria* group belonged to the genera *Klebsiella* (61%), *Pseudomonas* (4%), *Citrobacter* (4%) and *Acinetobacter* (1%). The *Betaproteobacteria* were represented by a single genus, *Achromobacter*. Members of the phylum *Firmicutes* were the second most abundant group in the rhizosphere (R fraction) and all the isolates belonged to the genus *Bacillus* (Table 3 and Fig. S2).

**Table 4:** Diversity indexes of the microbial collection.

Diversity Index(OTUs 99%)	E	R	S	B
Taxa	9	12	15	10
Individuals	77	74	73	75
Dominance	0.262	0.252	0.240	0.372
Shannon	1.586	1.799	1.930	1.429
Simpson	0.738	0.748	0.760	0.628
Evenness	0.543	0.504	0.459	0.417

The indexes were calculated for the sequences of bacterial strains isolated from the different fractions of the pepper root system and the non-cultivated arid soil. Sequences have been grouped in OTUs based on nucleotide similarity at 99%.

Similarly to the rhizosphere, in the S fraction two dominant phyla were detected: *Gammaproteobacteria* (71%) and *Firmicutes* (28%), with 4 genera in total: *Klebsiella* (44%), *Bacillus* (27%), *Citrobacter* (16%) and *Raoultella* (7%). The non-cultivated arid root-free soil was affected by the lowest Shannon and Evenness indices, pointing to a highly stable microbial community. The isolates from the B fraction were affiliated to three phyla: *Actinobacteria* (60%), *Firmicutes* (35%) and *Gammaproteobacteria* (5%). The genus *Cellulosimicrobium* was the major taxon (57%), followed by the genera *Bacillus* (39%), *Rhodococcus* (3%) and *Klebsiella* (1%) (Table 3 and Fig. S2).

A comparative analysis highlighted that strains of *Paenibacillus* (30%) were isolated only from fraction E. While members of *Gammaproteobacteria* were retrieved only in soil fractions, some genera showed a specific distribution: *Pseudomonas* was found only in R and S fractions; *Acinetobacter* only in R, strains of the *Raoultella* genus only in S and bacteria affiliated to *Cellulosimicrobium* and *Rhodococcus* only in B (Table 3).

### Plant Growth Promoting Activities and Tolerances to Abiotic Stress of the Isolates

The potential functionality of pepper associated isolates to sustain plant growth under drought was assessed by a large screening for PGP abilities in relation to drought tolerance, and the resistance to abiotic stresses occurring in arid soils (Table 5). We assessed whether PGP abilities are differentially distributed in the different microhabitats of the pepper root system. All the fractions demonstrated to be colonised with a similar frequency by potential beneficial strains, even though in the non-cultivated arid soil PGP traits were less abundant (Table 5). While none of the isolates showed all the assayed PGP activities, 31,7% and 22,5% of strains presented respectively four and five PGP activities (Fig. S1). All the isolates presented the potential to adapt to unfavourable environmental conditions of arid soils, showing a certain halotolerance, resistance to low water availability and to variable temperature range (Table 5). Similarly, bacteria isolated from the E, R and S plant-associated fractions exhibited a large number of PGP traits compared to isolates from arid non-cultivated root-free soil (B fraction) (Table 5). Nevertheless, some abilities like nutrient supply (phosphate solubilisation, siderophore release), are more frequent in soil bacteria, while auxin synthesis, directly affecting plant hormone homeostasis, was primarily presented by endophytes (Table 5). PGP traits distribution among the different bacterial genera revealed that the *Bacillus* and *Klebsiella* showed a predominant role, even though other genera less frequently isolated, like *Pseudomonas*, *Raoultella* and *Paenibacillus*, exhibited a higher number of PGP potential activities (Table 6 and Table S2).

**Table 5:** PGP potential of the microbial collection.

Isolationmedia	Plantfraction(N° ofisolates)	PGP activities and Tolerance to Abiotic stress (%)													
		Aux.	P sol.	EPS	Sid.	NH_3_	Prot	5%NaCl	8%NaCl	10%NaCl	10%PEG	20%PEG	4°C	42°C	50°C
ACCd(Haplotypes)	E (5)	80	20	20	20	60	20	20	20	20	100	60	0	100	20
	R (8)	38	88	63	75	88	0	75	0	0	100	100	88	100	0
	S (6)	83	100	83	100	100	0	100	0	0	100	100	67	100	0
	B (5)	20	100	40	20	100	40	100	0	0	100	100	20	100	0
R2A/KB	E (24)	92	58	8	29	100	83	88	75	17	100	100	4	92	0
	R (24)	79	50	17	33	100	92	96	79	42	100	100	8	83	29
	S (24)	92	42	29	17	92	79	88	58	33	100	100	13	96	46
	B (24)	92	50	13	8	100	63	83	67	42	100	100	0	96	25

Percentage distribution of plant growth promoting activities and tolerance to abiotic stress among the isolates of the bacterial collection obtained from the different fractions of the pepper root system and the non-cultivated arid soil.

Auxin  =  auxin production; P Sol.  =  inorganic phosphate solubilization; EPS  =  exopolysaccharide release; Sid. = siderophores production; NH_3_  =  ammonia production; Prot.  =  protease activity; PEG = poly-ethylen-glycol.

**Table 6:** Bacterial genera distribution of the PGP potential.

Genus	N° ofIsolates	PGP activities and Tolerance to Abiotic stress (%)													
		Auxin	P sol	EPS	Sid	NH_3_	Prot	5% NaCl	8% NaCl	10% NaCl	10% PEG	20% PEG	4°C	42°C	50°C
*Achromobacter*	1	0	0	0	0	100	0	0	0	0	100	100	100	100	0
*Acinetobacter*	1	0	100	0	0	0	0	0	0	0	100	100	100	100	0
*Bacillus*	92	88	48	18	18	98	79	89	70	35	100	100	43	94	27
*Cellulosimicrobium*	2	0	100	50	0	100	0	100	0	0	100	100	0	100	0
*Citrobacter*	2	0	100	0	100	100	0	100	0	0	100	100	50	100	0
*Klebsiella*	8	75	100	100	100	100	0	100	0	0	100	100	62	100	0
*Lysinibacillus*	1	100	0	0	0	1	0	0	0	0	100	100	0	100	0
*Paenibacillus*	3	100	33	0	0	33	0	0	0	0	100	33	0	100	0
*Pseudomonas*	6	100	83	0	50	100	83	83	67	17	100	100	33	50	0
*Raoultella*	3	100	100	100	100	100	0	100	0	0	100	100	100	100	0
*Rhodococcus*	1	0	100	0	0	100	100	100	0	0	100	100	100	100	0

Percentage distribution of plant growth promoting activities and tolerance to abiotic stress according to the genera of the bacterial collection obtained from the pepper root system and the non-cultivated arid soil.

Auxin  =  auxin production; P Sol.  =  inorganic phosphate solubilization; EPS  =  exopolysaccharide release; Sid. = siderophores production; NH_3_  =  ammonia production; Prot.  =  protease activity; PEG = poly-ethylen-glycol.

### *In vitro* Rhizoplane Colonization

**Figure 3:**
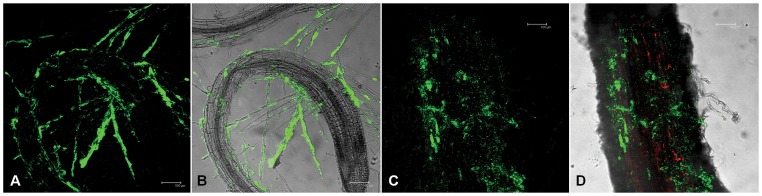
Rhizocompetence of *gfp*-labelled bacteria on different plant models. Plant root colonization experiments performed with a *Klebsiella pneumoniae* strain isolated from the pepper rhizosphere genetically labeled with a *gfp*. (A) and (B) colonization of *Arabidospis thaliana* rhizoplane; (C) and (D) colonization of the pepper rhizoplane. Red spots represent root autofluorescence as acquired through the TRICT filter. The scale bars of the different images in the figure correspond to 100 µm.

To assess the ability of soil bacteria to adhere and colonize the rhizoplane, an adhesion assay was performed *in vitro* on *Arabidopsis thaliana* roots by taking advantage of a *gfp*-labelled bacterium. Root colonization is a key requirement to ensure an intimate association with the plant and thus a support against water stress. Of the different strains assayed for transformation with plasmids carrying a *gfp* (Green Fluorescent Protein) cassette, we succeeded in transforming a *Klebsiella pneumoniae* strain. The *gfp*-tagged isolate was used to track the bacterial adhesion on *Arabidopsis* and pepper root system. After 15 h of exposure to the *gfp*-tagged bacterial suspension, confocal microscopy analysis revealed that *Arabidopsis* primary root and root hairs were massively colonized by *gfp*-tagged cells. The *gfp*-labelled bacterium completely enwrapped root hairs, with an adherence profile that was adapted to the root hair morphology (Fig. 3A–B). In pepper the strain was massively detected on the rhizoplane but only few cells were found on root hairs (Fig. 3C–D), suggesting a differential colonization profile according to the model plant.

### Selection of Rhizobacteria for Plant Growth Promotion under Drought Stress

**Figure 4:**
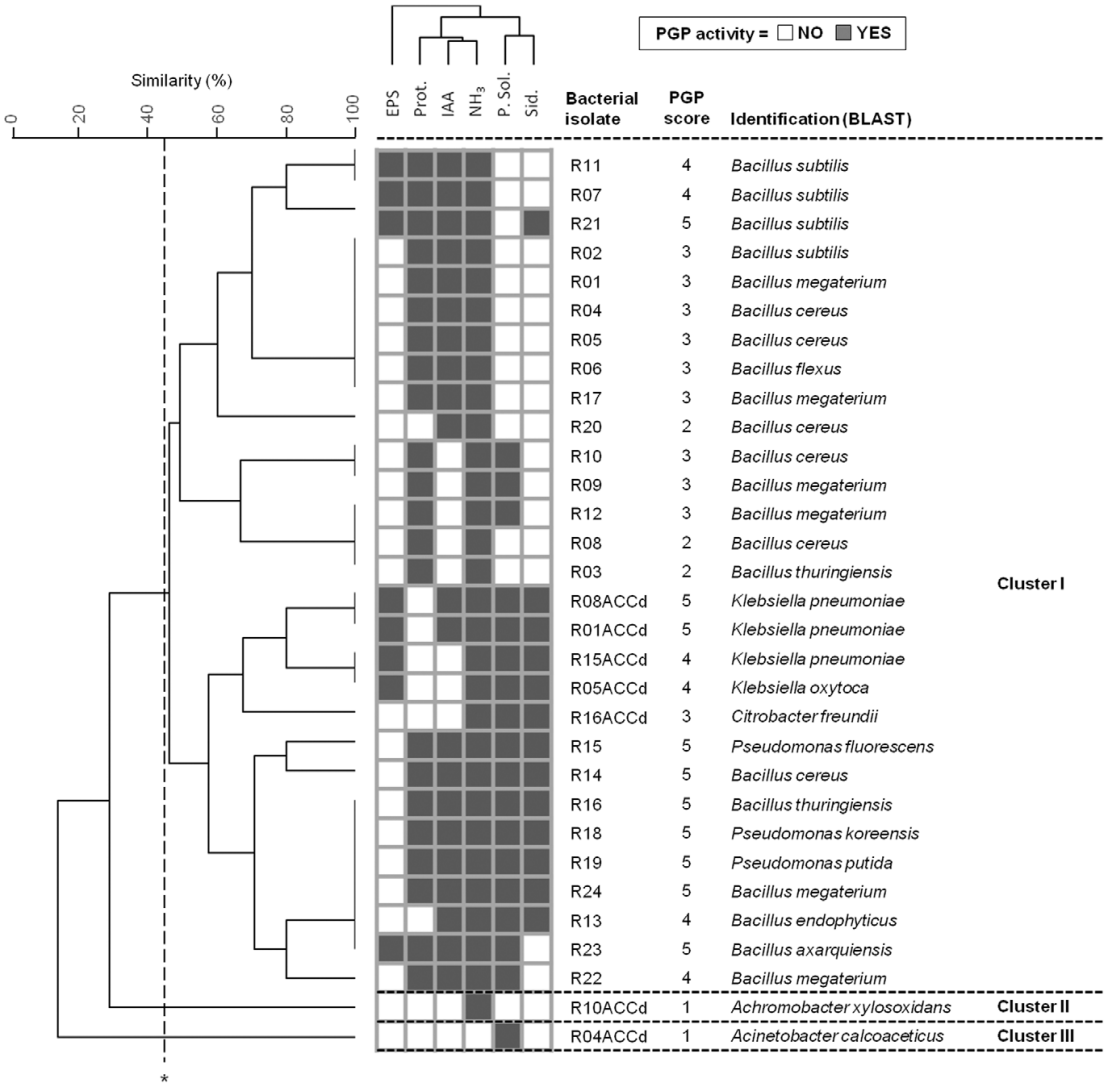
Analysis of the PGP potential of pepper associated rhizobacteria. Cluster analysis of the distribution of PGP activities in the rhizobacterial collection, according to Pearson correlation coefficient. Total PGP potential is indicated as a score value resulting from the sum of the number of the different PGP abilities exhibited by each strain. Cluster group were defined based on a cluster cutoff value of 42% of similarity.

Rhizobacteria were evaluated for the capability of promoting plant growth under water stress. A cluster analysis performed by combining the rhizobacteria PGP phenotypic traits (Fig.4) grouped the strains in three major clusters. Cluster I is the largest and summed *Bacillus* spp., *Klebsiella* spp. and *Pseudomonas* spp. The great majority of bacteria exhibiting ACCd activity were in this cluster that, moreover, included the strains with the highest number of potential PGP abilities. Clusters II and III displayed only one strain, respectively an ACCd-producing *Achromobacter xylosoxidans* and an *Acinetobacter calcoaceticus*. Both isolates exhibited just one PGP trait (Fig. 4). Consistent with ACCd activity in lowering plant ethylene under abiotic stress conditions, ACCd-producing rhizobacteria from the three clusters were selected to be further assayed in their ability to sustain plant growth *in vivo* under drought. These isolates were affiliated to genera *Citrobacter* (R16ACCd), *Klebsiella* (R01ACCd, R05ACCd, R08ACCd and R15ACCd), *Achromobacter* (R10ACCd) and *Acinetobacter* (R04ACCd), with *Klebsiella* spp. as the most frequent, as showed in the phylogenetic tree (Fig. 4 and S3).

### Plant Growth Promotion of Rhizobacteria Associated to Pepper Plants under Water Stress

**Figure 5:**
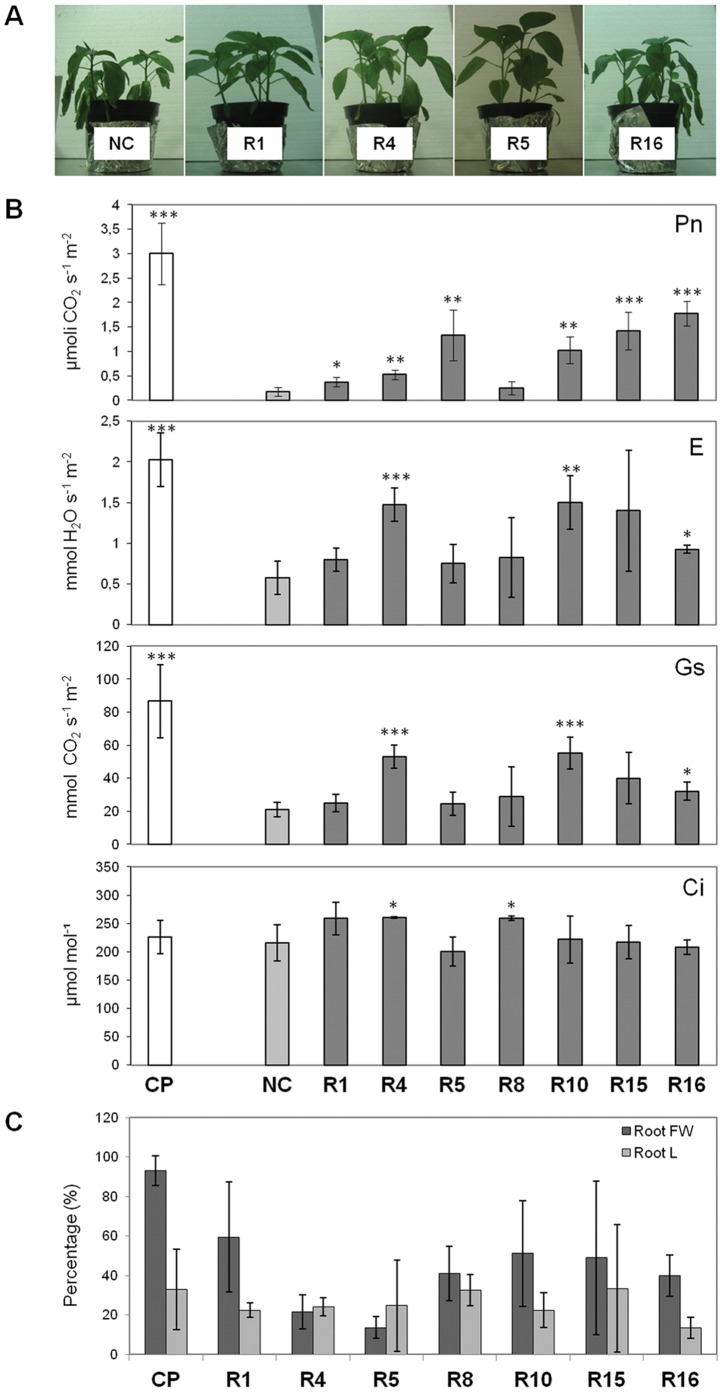
Rhizobacteria increased plant resistance to drought stress. Abbreviations for the figure: CP, (positive) abiotic control, irrigated at the water holding capacity of the soil along all the experiment; NC, (negative) abiotic control, subjected to drought by interrupting water supply for 12 days. (A) Representative images of plants exposed to rhizobacteria compared to untreated plants eight days after the induction of drought. (B) Leaf physiological parameters in treated and untreated plants eight days after the induction of drought.Abbreviations: Pn, net photosynthesis; E, evapo-transpiration; Gs, stomatal conductance; Ci, internal carbon dioxide (CO_2_). Student t-test was adopted to statistically analyse the data. *:p≤0,05; **:p≤0,01; ***:p≤0,001. The data reported in the graphs are representative of one replicate experiment. (C) Percentage increase in root fresh weight (FW) and root length (L) of water stressed plants, compared to the abiotic stressed control, set as 0%.

Well irrigated pepper seedlings inoculated or not with the rhizobacterial suspensions were suddenly exposed to a twelve days period of water stress. After eight days of water stress, control plants were severely affected, whereas plants exposed to ACCd-producing rhizobacteria exhibited a higher shoot turgor (Fig. 5A). Pepper plants inoculated with ACCd rhizobacteria R4, R10 and R16 showed net photosynthesis (Pn), evaporation/transpiration (E), stomatal conductance (Gs) significantly higher than untreated plants (NC), while R1, R5 and R15 strains positively affected water-stressed plants only at a photosynthetic level (Fig. 5B). At the end of the twelve days drought period, three days of re-watering were applied and plants were carefully harvested for biomass and length measure analysis. All plants exposed to the selected bacteria exhibited a more robust root system with a quantitative effect depending on the strain (Fig. 5C). A similar increase of about 20% in root length was observed both in non-stressed plants and in those inoculated with rhizobacteria respect to the plants exposed to drought (Fig. 5C). Root fresh weight in the inoculated plants showed a 40–60% increase depending on the bacterial strain, compared to the non inoculated stressed control plants (Fig. 5C).

## Discussion

The traditional management of agriculture in arid ecosystems is essential to preserve land from soil degradation and maintain food production ensuring a sustainability and preserving soil biodiversity [17]. A signature feature of arid and semi-arid lands is plant patchiness with scattered plant clumps dispersed in a bare landscape [18]. While the structure of the microbiota under and inter desert shrubs and canopies has been largely investigated [19], little attention was paid to the effect of desert farming on the structure and functionality of the microbiomes associated to plant root system. Recently, in a farm located at north-east Cairo, Egypt, Köberl et al. [4] reported higher biodiversity indices in cultivated fields than in the desert soil and the enrichment in bacteria with antagonistic activity against plant pathogens. Similarly, in cultivated fields at north-west Cairo, we found dramatic changes in the structure and activity of the bacteria associated to pepper root system compared to non-cultivated soil. A strong rhizosphere effect in terms of higher bacterial densities and species richness was observed in the soil fractions more closely associated to the root system, the R and S fractions, compared to the bulk root-free soil, whereas the endophytic fraction showed the lowest values, presumably because root tissues selected specific bacterial colonizers [20]. A certain variability was detected among endosphere replicates that could originate from multiple factors, including: microvariability in the soil field [20], [21], [22], [23], plant physiological condition [24], [25], growth stage [22], extent of root exudation [26], bacteria inter-species interactions and even random events [20]. Despite the sampled plants were coeval, the variability of the field conditions may have influenced the plant physiological state preventing to exclude a certain effect on the endosphere composition. Which combination of driving forces has determined the differences in the three replicates remains unresolved, however, the difference of the endosphere microbiome from the microbiome of the rest of the root system was clearly evident. This differential distribution is presumably triggered by the burst of microbial biomass that can use the organics rhizodeposed by the root determining the realization of a “resource island” effect typical of desert ecosystems where plant growth and interaction with the soil microbiome locally improves soil properties that in turn sustain the overall soil activity and biotic diversity [19]. Such a repartition of bacteria abundance and diversity observed in the pepper root system found analogies in other plant cultivated in arid soils, such as sugarcane [27], bamboo [28], chick pea [29], and olive tree (Marasco et al., unpublished data).

The distribution of the bacterial genera reflects adaptation to the different microhabitats. The *Bacillus* genus was isolated in all the pepper fractions, with higher prevalence in the endosphere. Garbeva et al. [30] showed that the majority of Gram-positive bacteria in soils under different types of management regimes (permanent grassland, grassland turned into arable land and arable land), were putative *Bacillus* species. *Bacillus* spp. are also commonly found in arid land as a consequence of their ability to form endospores that allow bacterial survival for extended time periods under adverse environmental conditions [31]. *Bacillus* and related genera have been already reported to be associated to and promote the growth of a wide range of plants [32].

In our study *Paenibacillus* and *Lysinibacillus* genera were isolated only from pepper root endosphere. *Paenibacillus* is a common soil bacterium that has been described to present PGP properties. In particular, *P. polymyxa* has multiple plant beneficial activities, such as nitrogen fixation, soil phosphorus solubilisation and production of exopolysaccharides, hydrolytic enzymes, antibiotics and cytokinin [33]. Inoculation of *Arabidopsis* and wheat with a *P. polymyxa* strain, isolated from rhizosphere of wild barley in northern Israel, resulted in enhanced drought tolerance [34]. The presence in the pepper root tissue of *Lysinibacillus* spp., a poorly studied genus isolated also from rather different plants such as bamboo [28], citrus [35], tomato [36], medicinal plants [37] and halophytes [38] needs a clarification of its role in the microbe-plant interaction.

The pepper root systems in the arid Egyptian soil showed to host endophytes only within the *Firmicutes* class, while previous studies for endophytes in both herbaceous and arboreal plants reported a diverse array of bacterial species, including members of *Acetobacter, Arthrobacter, Bacillus, Burkholderia, Enterobacter, Herbaspirillum, Serratia* and *Pseudomonas* [16], [39], [40], [41], [42]. In sweet pepper, culturable endophytes were assigned to high-G+C Gram-positive *Microbacterium*, *Micrococcus* and *Rhodococcus* but also to *Firmicutes* of the *Bacillus* and *Staphylococcus* genera. In other studies, the variability in the diversity of culturable endophytic bacteria has been associated to different selective pressures determined by the different pepper cultivars [43].

In all the soil fractions strains belonging to *Gammaproteobacteria* were predominant with many of the isolates assigned to the *Enterobacteriaceae* family. It comprises many species with enteric habitat, which origin could be attributable to the low hygienic quality of the irrigation water. The decline in the availability of pristine freshwater for irrigation due to allocation to urban and/or industrial supply, often results, especially in arid and semi-arid regions, in the intensive use of low-quality water to satisfy the increasing demand for irrigation. Representative species of *Enterobacteriaceae* genera, especially *Klebsiella*, *Enterobacter*, *Citrobacter*, have been isolated from different plant species grown in arid lands [44], [45], [46], [47]. In non-cultivated soil not subjected to irrigation and soil amendment, *Enterobacteriaceae* decreased in favour of *Actinobacteria*, with the prevailing genera *Cellulosomicrobium* and *Rhodococcus*. Together with *Bacillus* spp., *Actinobacteria* can survive as spores under adverse environmental conditions, hence making them typical desert taxa [4], [48].

The PGP features of bacteria associated to the pepper root system indicated that arid soils are excellent reservoir of bacteria responsible for the efficient functioning of the plant-soil ecosystem services. Twenty three percent of the assayed isolates exhibited multiple PGP activities, which may promote plant growth directly, indirectly or synergistically. Moreover, *gfp* labelling of a rhizobacterium demonstrated a versatile colonization capabilities being capable of colonizing the roots of two different plant models, as previously described for other bacteria [49].

A relative large range of PGP activities was recorded for bacteria isolated from non-cultivated soil, with 38% of isolates displaying more than 4 PGP activities, compared to 58% for isolates from R and S fractions. As the boundary between the non-cultivated soil and the desert areas around the farm was labile, we can perceive the still unexplored biotechnological potential of arid lands. Chanal and colleagues [50] found new radiotolerant bacterial species in Tataouine desert and recently *Ramlibacter tataouinensis* genome annotation revealed unexpected adaptation mechanisms to hot and dry environments, including sensitivity to light and to water availability at the dew time [50], [51]. A survey of PGP bacteria associated to *Hordeum spontaneum* in the “Evolution canyon” in Israel reported a significant higher population of osmotic tolerant, phosphate solubiliser, EPS producer and ACCd bacteria in the stressful sunny site than in the shadowed site [34]. According to these data it was assumed that the foundations for the adaptability to the harsh conditions of agriculture in arid lands are based on the co-evolution of the association between plant and microbes under harsh environmental conditions [34], [52]. In our bacterial collection from the pepper root system 88% of isolates showed multiple PGP activities and were able to grow at high temperature and at low water potential indicating that they can be active and hence express their PGP features *in vivo* under water stress conditions.

Drought is responsible for the weakening of ecosystem services, even at temperate latitudes. In 2003, a summer heat wave along with a prolonged drought event in Europe caused a reduction of 36% in the net productivity of maize in the Po valley in Italy and dramatically compromised agricultural production in France (−17%) and Eastern Europe (−20%) [53]. In Egypt agriculture strongly relies on the exploitation of the water from the Nile river, considering the limited availability of groundwater. In such general condition of water limitation it is supposed that other factors, like the root-bacteria association are selected for contributing to alleviate plant water stress. A candidate group of PGP bacteria that can have a potential protecting effect against water stress are ACCd rhizobacteria. ACCd bacteria are capable of lowering the concentration of ethylene that is overproduced in response to stressful conditions [54]. ACCd bacteria have been shown to recover plants from different stresses [55]. Different plant models have been successfully recovered from a variety of stressful conditions such as salinity [56] drought [57] and heavy metals [58] following the exposure to ACCd bacteria [59]. Hence, we have selected the collection of ACCd bacteria isolated from the pepper root system for assessing the capability of protecting the plant from drought and water stress.

Early responses to water stress include a decrease in photosynthesis efficiency [60]. Pepper plants treated with ACCd rhizobacteria recorded higher values for the photosynthesis processes and even a higher tissue turgor. These beneficial effects result in the increase of root biomass and length, up to 50% respect to non-inoculated plants. Although the rhizobacterial strains exhibited a variable extent in the improvement of plant drought tolerance, the most pronounced protection against drought was obtained with strains of the genera *Achromobacter*, *Klebsiella* and *Citrobacter*. Considering the root-colonization capacity of these genera it is conceivable that such protecting activity can be performed also in field conditions.

Desert bloom remains a general vision, although the real efficacy of PGP treatment of plants for desert restoration remains contradictory. A three-years field trial in the Sonoran desert with different tree species exposed to AM fungi and *Azospirillum brasilense* to restore degraded lands showed that the treatments were only partially successful. Positive results were obtained only with autochthonous leguminous trees while other combination of tree-inoculant-amendment resulted in small negative or no effect at all [1]. Our data indicate that consolidated traditional desert farms represents “resource islands” were topsoil is preserved from destruction by the wind or other soil erosion agents, contributing to act as a sink for organic matter and beneficial microbes. Desert farming remains a bulwark for protecting soil fertility in desert ecosystems and an effective strategy for enriching plant growth promoting microorganisms capable of directly protecting plants from drought stress.

## Materials and Methods

### Site Description and Sampling

Plant and soil samples were collected in a cultivated field in a private traditional farm located in the north-western desert region in Egypt, near El-Tawheed Village. The permission for sample collection was obtained by the Department of Horticulture of the University of Ain Shams, Egypt. Crop irrigation was performed using the water from the Nile river and groundwater. Four different fractions were collected in triplicates: E (endosphere), R (rhizosphere) and S (root surrounding soil) of *Capsicum annum* L. plants and B (bulk soil) as control. Intact roots were collected after plant eradication, with soil particles still adhering on the rhizoplane (E+R fractions). Soil around the collected roots and not attached to plant root system was sampled (S fraction). Uncultivated soil (B fraction) was kept as control at 4 m far from the cultivated field, in an area not subjected to irrigation and that was not cultivated in the last years. All soils and roots samples were collected under sterile condition using sterile tools. Recovered samples were stored at 4°C for microbiological isolation or stored at −20°C for molecular analysis.

### PCR-DGGE Analysis of Pepper Associated Bacterial Communities

Primers 907R and 357F with a GC-clamp were used in this study for the amplification of bacterial 16S rDNA genes [61]. PCR reaction was performed in 0.2 ml tubes using 50 µl reaction volume. The reaction mixture contained the diluted buffer 1 X, 1,5 mM MgCl_2_, 5% of DMSO, 0,12 mM of a mixture of dNTPs, 0,3 µM of each primer, 1 U Taq polymerase, and 10 ng of template. If necessary, DNA was properly diluted. Cycling conditions used to amplify the 16S rDNA gene fragment were 94°C for 4 min, followed by 10 cycles of 94°C for 0.5 min, 61°C for 1 min, and 72°C for 1 min; followed by further 20 cycles of 94°C for 0.5 min, 56°C for 1 min, and 72°C for 1 min; and a final extension at 72°C for 7 min. 2 µl of the PCR products were visualized by electrophoresis in 1.5% agarose gels stained with ethidium bromide prior to DGGE. For DGGE analysis, 100–150 ng of the PCR products generated from each sample were separated using polyacrylamide gel (8% of a 37∶1 acrylamide–bisacrylamide mixture in a Tris acetate EDTA (TAE) 1X buffer, 0.75 mm thick, 16×10 cm) with a 40–60% denaturant gradient). Gel was run overnight at 90 V in TAE 1X buffer at 60°C in DCode apparatus (Bio-Rad, Milan, Italy). The gel was stained with 1X Syber Green (Life Technologies) in TAE buffer and the gel was scanned with gel photo GS-800 system.

The DGGE bands were excised from the gel using a sterile cutter and eluted in 50 µl water at 37°C for 6 hours. The reamplification of DNA eluted from DGGE bands was performed using 907R and 357F primers without the GC-clamp, using the following protocol: 95°C for 5 min, 30 cycles of 95°C for 1 min, 61°C for 1 min, and 72°C for 1 min and a final extension at 72°C for 7 min. PCR products were checked by electrophoresis in 1% agarose gel. The sequencing service was performed by Macrogen Inc. (Korea). The band profile of fragments in the DGGE gel was converted in line plots with ImageJ software [62], and the x/y values obtained were imported into an Excel file. The matrix of x/y values of rRNA 16S line profiles was subjected to cluster analysis using the Pearson correlation coefficient. The multivariate analysis were conducted using XLSTAT software (vers. 7.5.2 Addinsoft, France).

### Isolation of Bacteria, Media and Culture Condition

R fraction, the soil particles tightly adhering to the rhizoplane, were separated from the root tissue (E) by applying the “pull and shake method”. Root surface was sterilized as described by Sun et al. [63] and the efficacy of the sterilization method was verified by plating the last wash water on King’s medium [64]. One gram of smashed E, R, S and B were suspended in 9 ml of sterile physiological solution (9 g/L NaCl) and shaken for 15 min at 200 rpm at room temperature. Suspension were diluted in 10-fold series and plated in triplicate onto KB medium and on R2A medium (Oxoid). After 3 days at 30°C, Colony Forming Units (CFU) per gram were determined. 12 colonies per medium per fraction were randomly selected and spread on the original medium for three times to avoid contamination risks. Moreover, 1 g of sample from each fraction was used as inoculum for ACC-deaminase enrichments as described by Penrose and Glick [65]. 50 colonies were randomly picked and propagated three times on PAF medium (10 g/L proteose peptone, 10 g/L hydrolyzed casein, 3 g/L MgSO_4_, 1,5 g/L K_2_HPO_4_, 10 mL/L glycerol and 15 g/L agar for solid medium). Pure strains were frozen in 25% glycerol at −80°C. A total of 299 isolates were collected and further characterized in this study.

### Phylogenetic Affiliation of Bacterial Strains

DNA was extracted from isolates by boiling lysis. The bacterial cells were resuspended in 50 µl of sterile TE (10 mM Tris/HCl, pH 8, 1 mM EDTA) in 1.5 ml tubes and incubated at 100°C for 8 min. After centrifugation (13000 g, 10 min), the supernatant containing the released DNA was stored at −20°C and used as template for PCR amplification. The bacterial collection originated from ACC enrichments was de-replicated by fingerprinting analysis of the rRNA 16S-23S Intergenic Transcribed Spacer (ITS) region. The ITS-PCR protocol was performed as described by Cardinale et al. [66]. The PCR products were separated by gel electrophoresis in 2% agarose gel and the fingerprinting profiles were visualized using Gel Doc system (Bio-Rad, Milan, Italy). Isolates which showed the same banding pattern were grouped in haplotypes, and for each haplotype a representative strain was selected for further analysis. Phylogenetic identification of isolates was performed by partially sequencing of the 16S rRNA gene, using universal primers 27F and 1492R. PCR products were checked by electrophoresis in 1% agarose gel. The sequencing service was performed by Macrogen Inc. (Korea). The sequences were compared with those deposited in the GenBank database, using the online software BLAST.

### Diversity and Phylogenetic Analyses

16S rRNA gene sequences were aligned using the ClustalX software [67] and the output file was used to define operational taxonomic units (OTUs) using DOTUR [68]. A quantitative matrix was created basing on the absence/presence of each polymorphic OTU calculated at 99% nucleotide similarity. Cluster analysis has been performed with the XLSTAT software using the Pearson correlation coefficient.

Number of Taxa, Shannon, Evenness, Simpson and Dominance indexes of the OTUs, defined at 99% of similarity, have been calculated using the PAST software [69].

The alignment of ACCd rhizobacteria sequences and the construction of the phylogenetic tree were performed using the neighbor-joining method [70] of MEGA version 4 [71].

### Nucleotide Sequence Accession Numbers

The partial 16S rRNA gene sequences (800–900 bp) from the isolates and the partial 16S rRNA gene sequences (500 bp) from the DGGE bands have been deposited in the GeneBank database from the accession numbers HE610774 to HE610892 and from HE856290 to HE856311 respectively.

### Evaluation of Direct and Indirect Plant Growth Promoting Activity and Tolerance to Abiotic Stresses

Indolacetic acid production was estimated following the protocol described by Brick et al. [72]. The mineral P-solubilizing ability of the strains was determined on Pikovskaya’s liquid medium amended with 0.5% [Ca_3_(PO_4_)_2_] as described by Mehta and Nautiyal [73]. Siderophore release was determined as described by Schwyn and Neilands [74]. Exopolysaccharides (EPS) production was estimated as described by Santaella et al. [75], using modified Weaver mineral media enriched with sucrose.

Ammonia production was evaluated as described by Cappuccino and Sherman [76]; protease production was determined in 5% agar skimmed milk [77]. Resistance to salt was assessed by adding 5–8–10% NaCl to the culture media and incubating the plates at 30°C for 5 days. Tolerance to osmotic stress was evaluated by adding to liquid media 10–20% of Poly-Ethylen-Glycol (PEG). The ability to growth at 4°, 42° and 50°C was verified in solid media by incubation at the indicated temperatures and the growth was qualitatively scored after 5 days of incubation.

### *In vitro* Bacterial Rhizocompetence Assay

The plasmid pHM2-*gfp* [78] was used to label R1-ACCd strain, affiliated to *Klebsiella* spp. Overnight culture of R1-ACCd was re-inoculated in fresh KB medium and the growth was monitored spectrofotometrically. When the culture reached 0.3 OD, 1 ml aliquot of cells were centrifuged (4000 rpm, 4°C) and washed twice with MilliQ water prior to be resuspended in 50 µl of MilliQ water and 10% glycerol. 30 µl of cells were used to be transformed by electroporation (Eppendorf 2510) with 50 ng of pHM2-*gfp* plasmid. Successful transformation was checked by growth on a selective medium (KB+50 µg/ml of kanamicin). To evaluate R1-*gfp* colonization ability, three-days *Arabidopsis thaliana* seedlings or seven-days *Capsicum annuum* L. seedlings were exposed to 10^8^ cells/mL. Seedlings dipped in sterilized water were used as negative control. After 15h, plants were rapidly washed to remove weakly bound bacteria and observed under a confocal laser scanning microscope (Leica TCSNT). Images were acquired using Leica Confocal Software, using BP530/30 GFP filter (exitation at 488 nm) and LP590 TRITC filter (excitation length at 568 nm). For pepper rhizocompetence analysis, images were acquired also using the TRICT filter to observe root architecture by exploiting root autofluorescence in this channel. The acquired images were analyzed by using the MBF ImageJ software.

### Plant Growth Promotion under Water Atress in Soil

Pepper seeds were sown in trays in wet agriperlite. After 1 week, uniform-sized seedlings were selected and planted in soil, three plants per 14-cm plastic pot. The seedlings were maintained in a growth chamber at a day/night temperature of 25/20°C with ∼100 µmol photons m^–2^ s^–1^ of light supplied for 12 h during the daytime. During the second week, the seedlings were fertilized once with a bacterial suspension at the concentration of 10^8^ cells/g of soils, while uninoculated plants were watered with tap water. One week after bacteria treatment, water was withhold for 12 days. A (positive) abiotic control, PC, was included and was properly irrigated all the experiment long. Seven-eight days after drought induction, physiological measures have been performed. To characterize photosynthesis performance, gas exchange measurements were taken with a portable photosynthesis system (CIRAS-2, PP System, USA). Measurements were taken on young, fully expanded, intact leaves of capsicum plants. Net CO_2_ assimilation rate, stomatal conductance and transpiration were assessed at a CO_2_ concentration of 400 µmol mol^−1^, 50% relative humidity, 28°C chamber temperature, 500 ml min^−1^ airflow and a photon flux density of 1500 µmol m^−2^ s^−1^. The instrument was stabilized according to manufacturer guidelines. After drought, water irrigation was resumed for three days and plants were harvested for biomass and length measures. Three independent experiments were performed with three replicate plants each. The statistical analysis was performed by analysing data by the T student test with (p<0,05).

## Supporting Information

Figure S1 **16S rRNA PCR-DGGE analysis of the bacterial communities in soil and endosphere of pepper plants.** (A) 16S rRNA gene PCR-DGGE profiles in different plant fractions (E, R, S and B) obtained from three replicate plants (indicated as 1, 2 and 3). Circles on the bands indicate the DNA fragments that were excised from the gel and successfully amplified and sequenced (see also Table 1). (B) Plot line conversion for each DGGE fingerprinting profile obtained using Image-J software.
(TIF)

Figure S2 **Diversity of culturable bacteria in pepper plant fractions.** Distribution of bacterial isolate genera associated to different fractions of the pepper root system compared to non-cultivated root free arid soil.
(TIF)

Figure S3 **Phylogenetic affiliation of pepper ACCd rhizobacteria.** Neighbour-joining phylogenetic tree based on 16S rRNA gene sequences of ACCd rhizospheric bacteria and their closest phylogenetic neighbours. Bootstrap values are indicated at nodes. Scale bar represents observed number of changes per nucleotide position.
(TIF)

Table S1 **Percentages of bacteria displaying PGP activities in different fractions of the pepper root system.** Isolates recovered from the pepper root system and its different fractions, presenting different numbers (from 0 to 6) of PGP activities.
(DOCX)

Table S2 **Distribution of the PGP potential according to the microbial genera.** The percentage of isolates displaying different numbers (from 0 to 6) of PGP activities are classified according to genus level, considering the whole microbial collection.
(DOCX)
