# Supplementary figures and images for: A Drought Resistance-Promoting Microbiome Is Selected by Root System under Desert Farming
Source: PLoS One. 2012 Oct 31;7(10):e48479. doi: 10.1371/journal.pone.0048479 (PMC3485337; doi:10.1371/journal.pone.0048479)

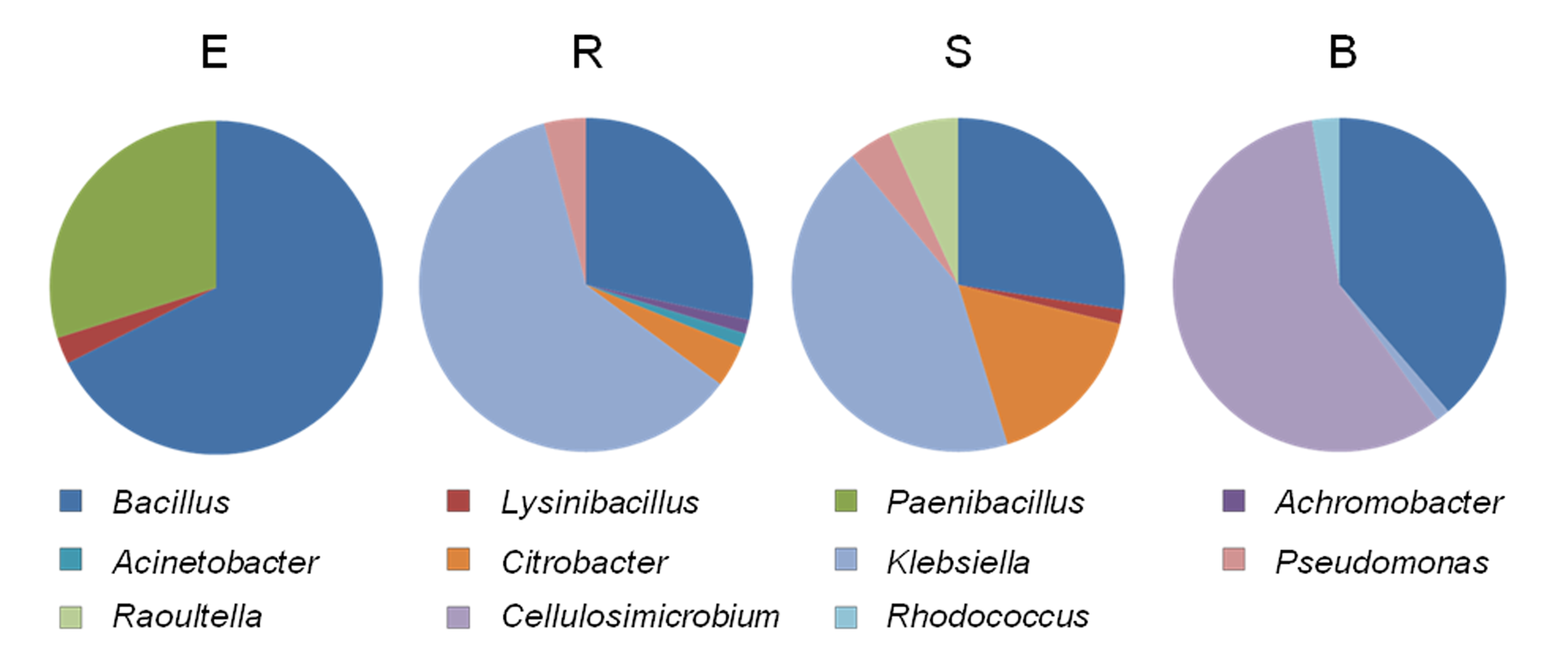

Supplement: Figure S2 — Diversity of culturable bacteria in pepper plant fractions. Distribution of bacterial isolate genera associated to different fractions of the pepper root system compared to non-cultivated root free arid soil. (TIF) [file pone.0048479.s002.tif]

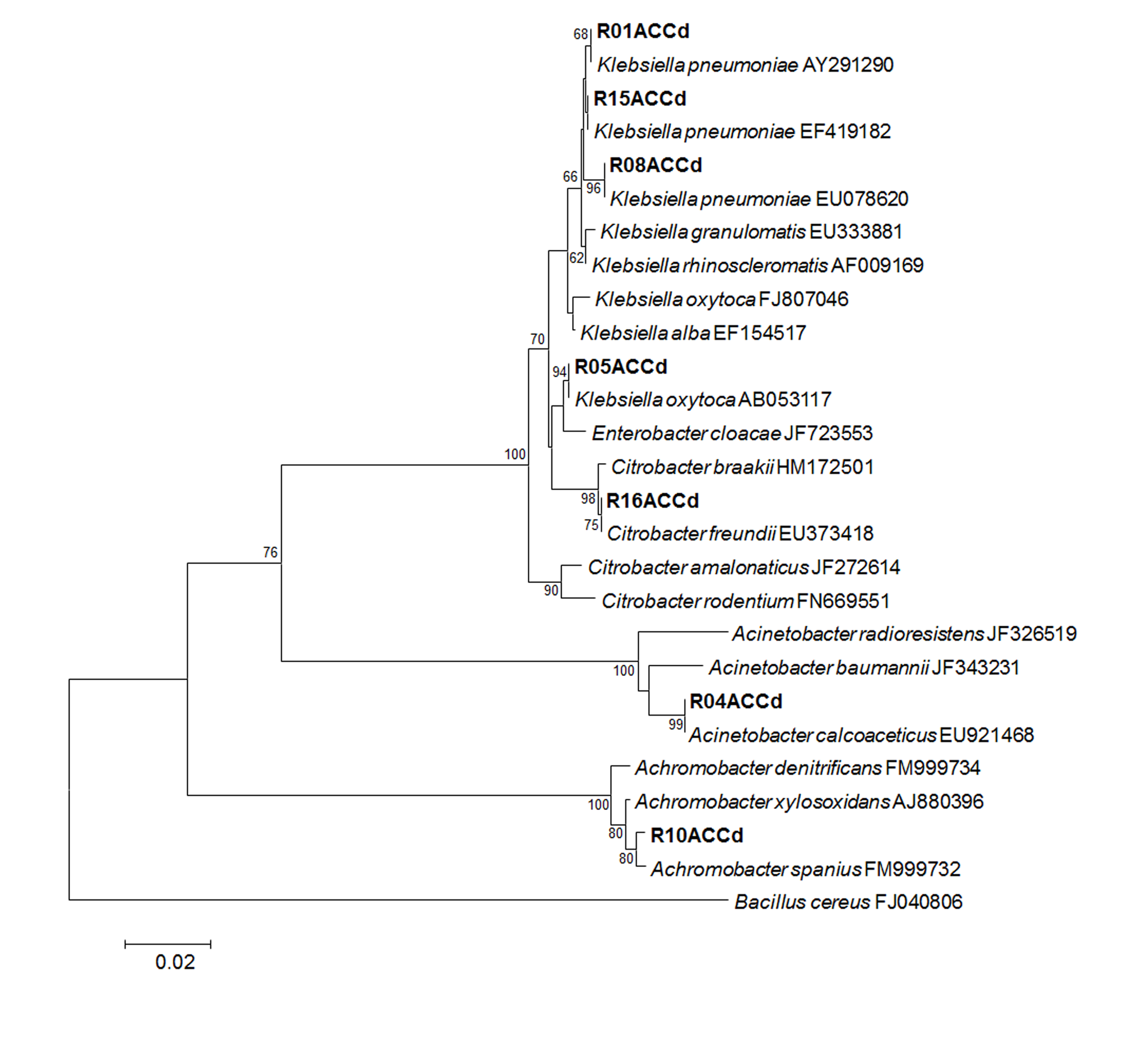

Supplement: Figure S3 — Phylogenetic affiliation of pepper ACCd rhizobacteria. Neighbour-joining phylogenetic tree based on 16S rRNA gene sequences of ACCd rhizospheric bacteria and their closest phylogenetic neighbours. Bootstrap values are indicated at nodes. Scale bar represents observed number of changes per nucleotide position. (TIF) [file pone.0048479.s003.tif]
