# Supplementary material for: A Drought Resistance-Promoting Microbiome Is Selected by Root System under Desert Farming
Source: PLoS One. 2012 Oct 31;7(10):e48479. doi: 10.1371/journal.pone.0048479 (PMC3485337; doi:10.1371/journal.pone.0048479)
Supplement: Table S1 — Percentages of bacteria displaying PGP activities in different fractions of the pepper root system. Isolates recovered from the pepper root system and its different fractions, presenting different numbers (from 0 to 6) of PGP activities. (DOCX) [file pone.0048479.s004.docx]

**Supplementary material Table 1. Percentages of bacteria displaying PGP activities in different fractions of the pepper root system.** Isolates recovered from the pepper root system and its different fractions, presenting different numbers (from 0 to 6) of PGP activities.

| **Isolates** | **N° of isolates (root system fraction from where have been isolated)** |  | **Percentages of isolates with n (0 to 6)**  **PGP activities** | | | | | | |
| --- | --- | --- | --- | --- | --- | --- | --- | --- | --- |
|  |  | **n =** | **0** | **1** | **2** | **3** | **4** | **5** | **6** |
| Total isolates | 120 |  | 0 | 5 | 11,7 | 29,2 | 31,7 | 22,5 | 0 |
| Isolates on ACCd enrichment medium | 5 (E) |  | 0 | 20 | 40 | 20 | 0 | 20 | 0 |
|  | 8 (R) |  | 0 | 25 | 0 | 12,5 | 25 | 37,5 | 0 |
|  | 6 (S) |  | 0 | 0 | 0 | 16,7 | 0 | 83,3 | 0 |
|  | 5 (B) |  | 0 | 0 | 20 | 60 | 0 | 20 | 0 |
| Isolates on R2A/KB medium | 24 (E) |  | 0 | 0 | 12,5 | 16,7 | 50 | 20,8 | 0 |
|  | 24 (R) |  | 0 | 0 | 12,5 | 37,5 | 16,7 | 33,3 | 0 |
|  | 24 (S) |  | 0 | 8,3 | 0 | 33,3 | 50 | 8,3 | 0 |
|  | 24 (B) |  | 0 | 4,2 | 20,8 | 33,3 | 33,3 | 8,3 | 0 |

E, isolates obtained from the interior of plant tissues; R, isolates obtained from the soil attached to the root; S, isolates obtained from the soil loosely attached to the root; B, isolates obtained from the root-free bulk soil.
