# Supplementary material for: A Drought Resistance-Promoting Microbiome Is Selected by Root System under Desert Farming
Source: PLoS One. 2012 Oct 31;7(10):e48479. doi: 10.1371/journal.pone.0048479 (PMC3485337; doi:10.1371/journal.pone.0048479)
Supplement: Table S2 — Distribution of the PGP potential according to the microbial genera. The percentage of isolates displaying different numbers (from 0 to 6) of PGP activities are classified according to genus level, considering the whole microbial collection. (DOCX) [file pone.0048479.s005.docx]

**Supplementary material Table 2. Distribution of the PGP potential according to the microbial genera.** The percentage of isolates displaying different numbers (from 0 to 6) of PGP activities are classified according to genus level, considering the whole microbial collection.

| **Microbial genera** | **N° of isolates** |  | **N° of strains with n PGP activity** | | | | | | |
| --- | --- | --- | --- | --- | --- | --- | --- | --- | --- |
|  |  | **n =** | **0** | **1** | **2** | **3** | **4** | **5** | **6** |
| *Achromobacter* | 1 |  | 0 | 100 | 0 | 0 | 0 | 0 | 0 |
| *Acinetobacter* | 1 |  | 0 | 100 | 0 | 0 | 0 | 0 | 0 |
| *Bacillus* | 92 |  | 0 | 3,3 | 12 | 30,4 | 38 | 16,3 | 0 |
| *Cellulosimicrobium* | 2 |  | 0 | 0 | 50 | 50 | 0 | 0 | 0 |
| *Citrobacter* | 2 |  | 0 | 0 | 0 | 100 | 0 | 0 | 0 |
| *Klebsiella* | 8 |  | 0 | 0 | 0 | 0 | 25 | 75 | 0 |
| *Lysinibacillus* | 1 |  | 0 | 0 | 100 | 0 | 0 | 0 | 0 |
| *Paenibacillus* | 3 |  | 0 | 33,3 | 33,3 | 33,3 | 0 | 0 | 0 |
| *Pseudomonas* | 6 |  | 0 | 0 | 0 | 33,3 | 16,7 | 50 | 0 |
| *Raoultella* | 3 |  | 0 | 0 | 0 | 0 | 0 | 100 | 0 |
| *Rhodococcus* | 1 |  | 0 | 0 | 0 | 100 | 0 | 0 | 0 |
